# Structure modeling to function prediction of Uncharacterized Human Protein C15orf41

**DOI:** 10.6026/97320630014206

**Published:** 2018-05-31

**Authors:** Md. Shakil Ahmed, Md. Shahjaman, Enamul Kabir, Md. Kamruzzaman

**Affiliations:** 1 Department of Statistics, University of Rajshahi, Rajshahi-6205, Bangladesh; 2 Department of Statistics, Begum Rokeya University, Rangpur-5400, Bangladesh; 3 School of Agricultural, Computational and Environmental Sciences, University of Southern Queensland, Australia; 4 Data Science for Knowledge Creation Research Center, Seoul National University, Korea

**Keywords:** Uncharacterized human protein C15orf41, Phylogenetic analysis, Protein domain, PTM sites, PPI networks

## Abstract

The dyserythropoietic anemia disease is a genetic disorder of erythropoiesis characterized by morphological abnormalities of erythroblasts. This is caused by human gene C15orf41 mutation. The uncharacterized C15orf41 protein is involved in the formation of a functional complex structure. The uncharacterized C15orf41 protein is thermostable, unstable and acidic. This is associated with TPD (Treponema Pallidum) domain (135 to 265 residue position) and three PTM sites such as K50 (Acetylation), T114 (Phosphorylation) and K176 (Ubiquitination). C15orf41 is paralogous to isoform-1 (gi|194018542|) and open reading frame isoform-CRA_c (gi|119612744|) of Homo sapiens located at chromosome 15. It interacts with the human ATP (Adenosine Triphosphate) binding domain 4 (ATPBD4) having similarity score 0.725 as per protein-protein interaction (PPI) network analysis. This data provides valuable insights towards the functional characterization of human gene C15orf41.

## Background

The human uncharacterized gene C15orf41 is located at chromosome 15 encodes a protein with two predicted helix-turnhelix domains. Mutations of this gene are found in the family of congenital dyserythropoietic anemia type-I [1]. This anemia disease description is an autosomal recessive blood disorder characterized by morphological abnormalities of erythroblasts, macrocytic anemia, secondary hemochromatosis and unproductive erythropoiesis. It is ccasionally associated with bone abnormalities, especially of the hands and feet (acrodysostosis), nail hypoplasia and scoliosis. Ultrastructural features include inter-nuclear chromatin bridges connected with some nearly erythroblasts. It is completely separated and an abnormal appearance (spongy or Swiss-cheese entrance) of the heterochromatin in a high proportion of the erythroblasts.

The structural and functional characteristics of proteins play the significant role in drug design and discovery. Investigations of these proteins characteristics experimentally in the wet lab are much laborious, time consuming and costly. The computational/statistical tools of bioinformatics reduce this cost and time significantly to characterize the uncharacterized proteins. These tools are widely used for homology modeling of sequence profiles and predicting the three-dimensional (3D) structure of the targeted protein. The homology modeling is utilized when the experimentally obtained structure is unavailable. It can provide a useful 3D model for the protein of interest that is related to at least one known protein structure. It is also used to predict the 3D structure of one or more proteins of known structure for a given protein sequence based on the primarily sequence alignment. The inclusive municipal sequences are increasing in some databases like SwissProt [2] and NCBI compared with the amount of experimentally determined structures deposited in the Protein Data Bank (PDB) [3]. Some gaps are created during multiple sequence alignment. The bioinformatics tools predict the 3D structures of protein by reducing those gaps to confirm the biological functions [4]. The in silico/computational prediction of 3D structures is validated experimentally in the wet lab using X-ray crystallography and NMR spectroscopy [5]. There are several new computational/statistical bioinformatics tools those are better than the classical homology search tools. Some functional clues of hypothetical proteins are investigated based on genomic context analysis [6]. The deep convolutional neural fields can be used for analyzing protein sequence profiling to predict the protein secondary structure for the conformation of its cellular functions [7]. The protein expression profile analysis also aids to understand the function of uncharacterized proteins [8]. An evolutionary characterization of uncharacterized bacterial proteins based on sequence profile analysis by computational approach is more rapid than experimental approach, which is very important for the discovery of drug targets and biological process [9, 10]. The quantitative analysis has been conducted for the understanding of human airway cilla protein functionality [11]. It is also useful to understand the functionality of unknown protein and its residue catalysis [12]. There are several other methods addressed in the literature for prediction of uncharacterized proteins functions [13, 14, 15, 16]. However, the characterization of this crucial uncharacterized protein C15orf41 was studied experimentally only in the wet lab. It would be interesting to study these properties computationally using bioinformatics tools to reduce the time and experimental cost. Therefore, in this study, an attempt is made to investigate the physiochemical properties and structural and functional characteristics of this protein using computational/statistical bioinformatics tools.

## Methodology

### Sequence Data

The target/query sequence (human uncharacterized protein C15orf41) was collected in FASTA format using the accession number Q9Y2V0 from the UniProt protein database (http://www.uniprot.org/uniprot/Q9Y2V0) [17]. Reference/template protein sequences were collected from the protein databases of NCBI (http://www.ncbi.nlm.nih.gov/) and SwissProt (http://www.expasy.org/sprot/) [2]. Protein sequences and their related information are publicly available in both databases.

### Homology Modelling

Homology modelling are used to identify the structure of the query protein sequence based on one or more known protein structures and on the production of an alignment that maps residues in the query sequence to residues in the template sequence. The quality of the homology model is associated with the quality of the sequence alignment approach and template structure. Homology model produces high-quality structural models when the target and template are closely related. Homology search of the target/query protein sequence with the reference/template sequences are performed using the online bioinformatics tool "BLASTp (Basic Local Alignment Search Tool of Protein)" of NCBI database [18].

### Physiochemical Properties

Various physiochemical properties of the query protein like the number of amino acids, molecular weight, hypothetical isoelectric point (pI), amino acid composition (%), number of positively (Arg + Lys) and negatively charged (Asp + Glu) residues, extinction coefficient, instability index, aliphatic index and Grand Average of Hydropathicity (GRAVY) are investigated using the online ExPASy's ProtParam tool (http://expasy.org/tools/protparam.html).

### Domain Coposition and PTM Site Prediction

A protein consists of one or more domains for functional activities in the cellular processes. Some domains show their functions regularly and some are active during the evolution only. The post translational modification (PTM) is the modification of amino acid covalent based on the protein sequence and it is important issues for regulating of biological and physiological functions in the cell [19]. The query/target protein domain composition and PTM sites prediction both are performed using the bioinformatics tool named 'SMART' (http://smart.embl-heidelberg.de/) [20].

### Multiple Sequence Alignment and Phylogenetic Analysis

The computational/statistical offline bioinformatics tool (ClustalW in MEGA5.0 [21]) is used to align the query sequence with the template/reference sequences quickly. This alignment is known as multiple sequence alignment (MSA). All aligned sequences are used to find the pattern/group of the query sequence using the neighbor joining (NJ) approach of phylogenetic analysis. All the similarities and dissimilarities of aligned sequences with query sequence are highlighted using the offline tool GENEDOC.

### Secondary Structural Prediction

The secondary structure of the query protein sequence is predicted using the online bioinformatics tool SABLE (http://sable.cchmc.org/) [22]. Then its 3D structure is predicted using the online tool SWISS-MODEL (https://swissmodel.expasy.org/).

### Function Prediction

Protein-protein interaction (PPI) networks analysis of a query protein with the template/reference proteins is important for more accurate prediction of its function. The PPI network is performed using online STRING (http://string-db.org/) bioinformatics tool [23].

### The Work Flow

**Figure 1:**
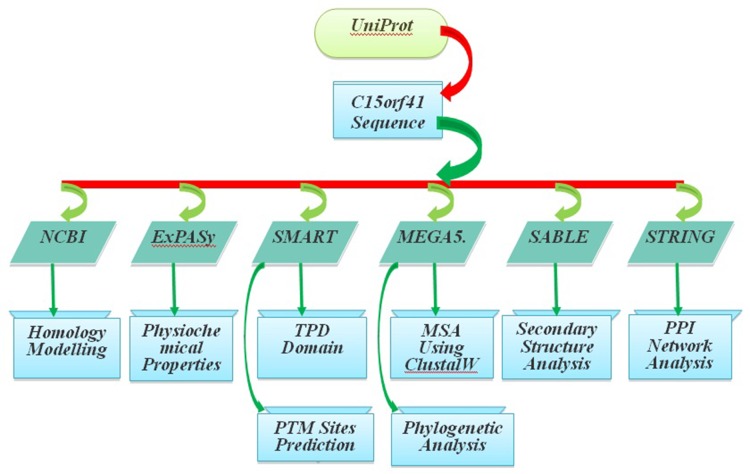
Flowchart of the study.

The detail workflow of this study is shown in Figure 1.

## Results & Discussion

**Figure 2:**
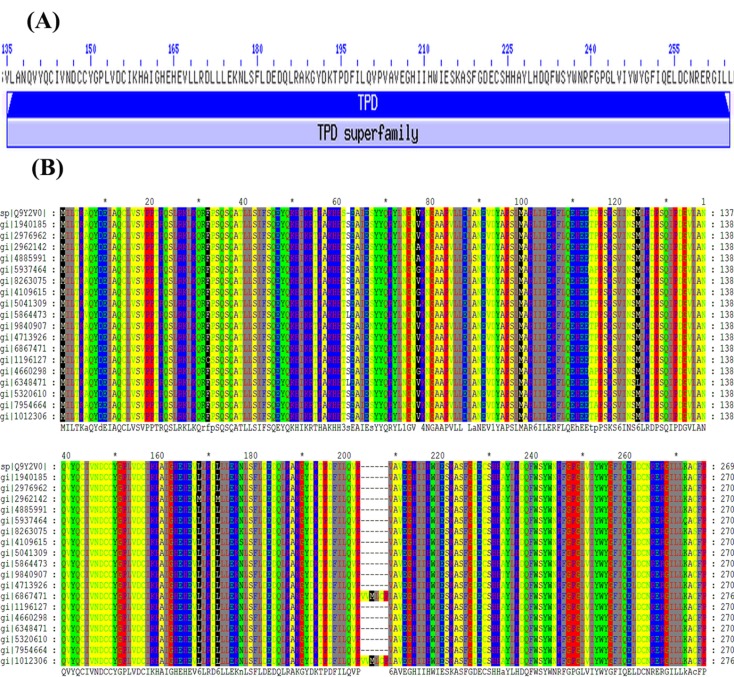
(A) Protein domain structure of uncharacterized human protein C15orf41 (135-265) and (B) Multiple sequence alignment of best homology (mostly conserved) protein with uncharacterized protein.

**Figure 3:**
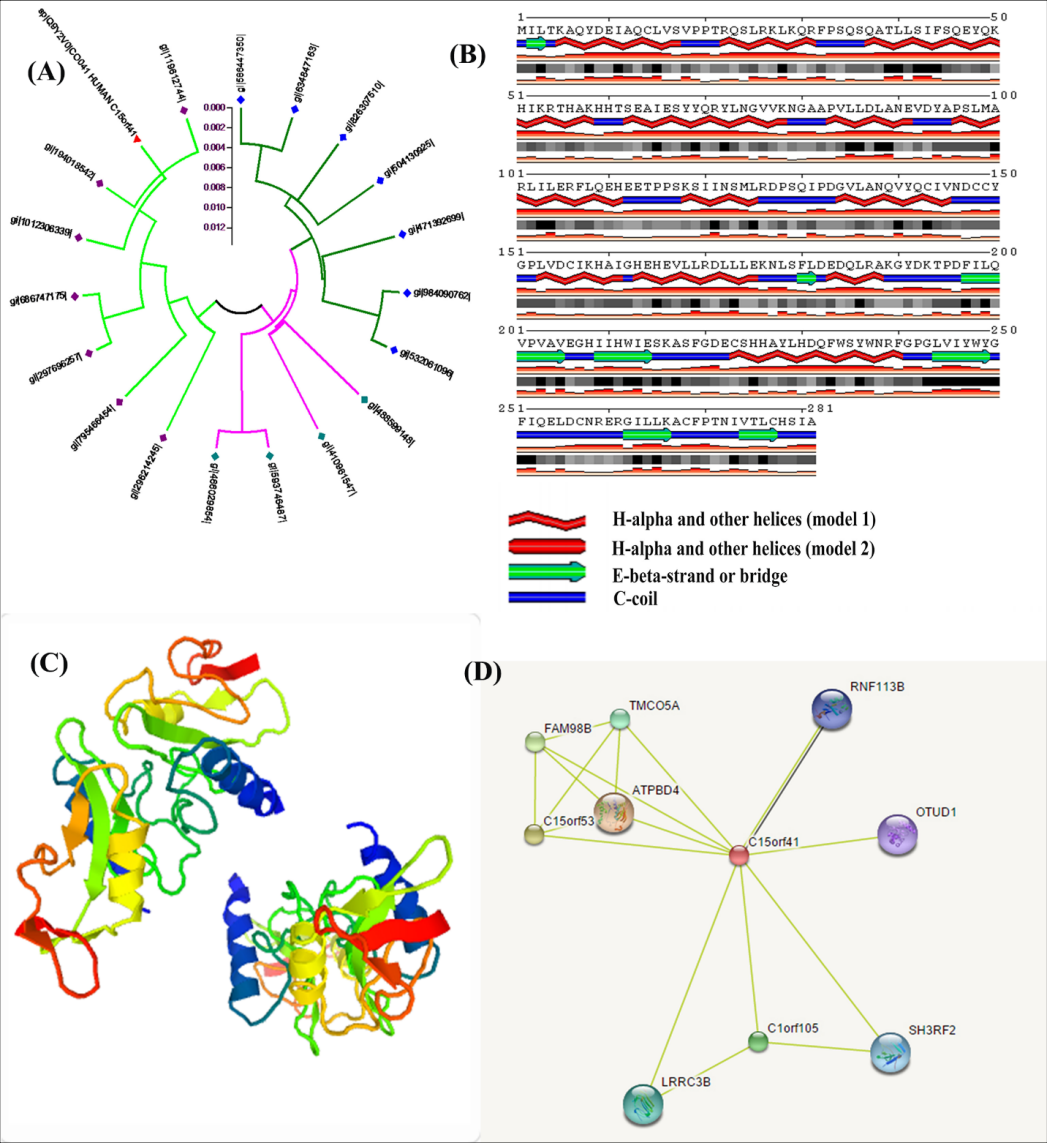
(A) Phylogenetic tree analysis of uncharacterized human C15orf41 and best homology protein, (B) Secondary Structure of human uncharacterized protein C15orf41 by SABLE, (C) Three (3) dimensional structure of uncharacterized protein C15orf41 has performed by the SWISS-MODEL (https://swissmodel.expasy.org/) and (D) Protein-Protein Interaction (PPI) network of uncharacterized protein C15orf41.

**Figure 4:**
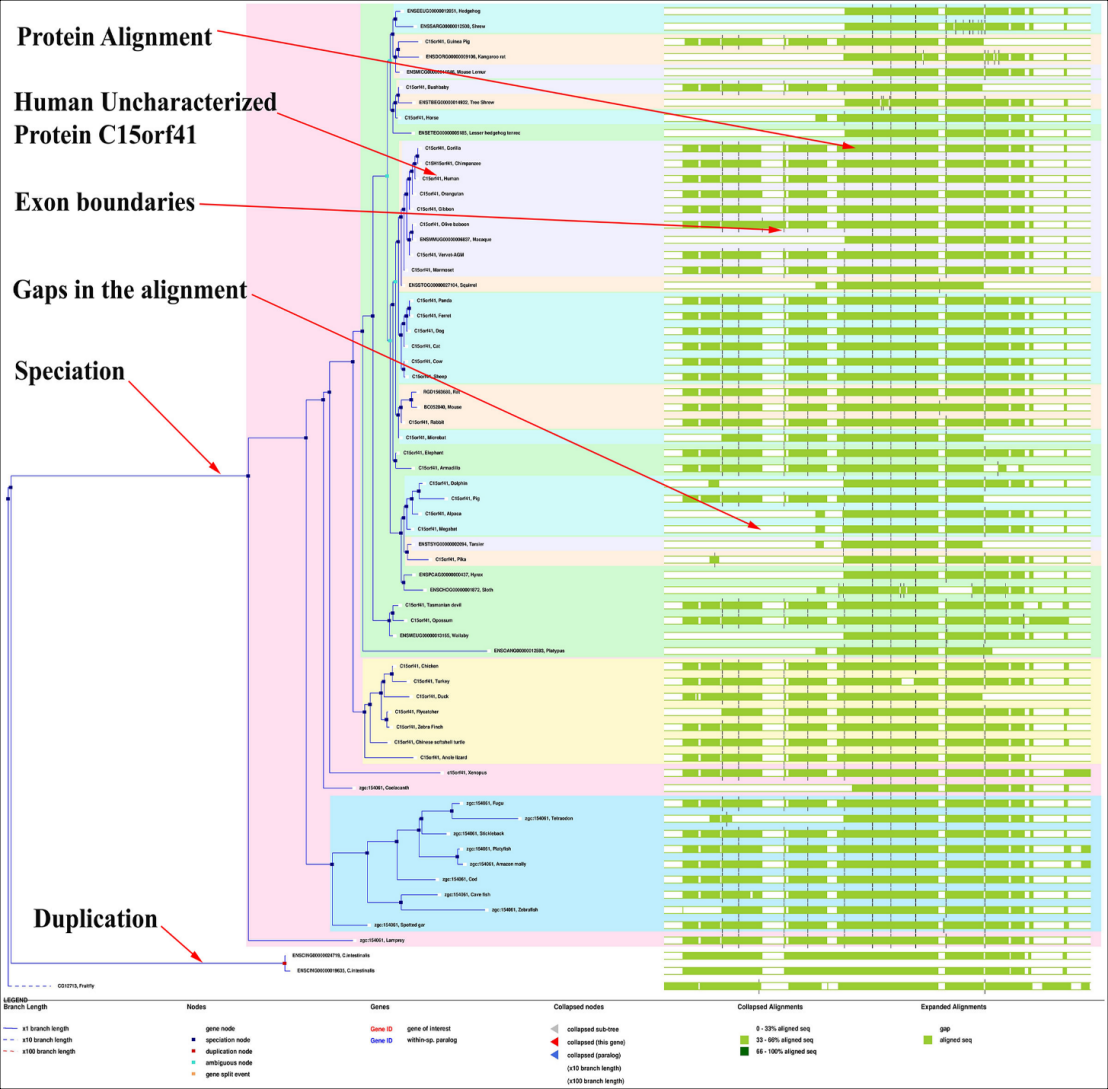
Phylogenetic gene tree and protein domain structure of human uncharacterized protein C15orf41 using online bioinformatics tool e!Ensembl (http://asia.ensembl.org/Help/View?id=137).

Structural and functional characteristics of human protein C15orf41 (query protein) was analyzed using the several online and offline bioinformatics tools. At first, we performed the homology modeling of query protein with the reference/template protein sequences using the BLASTp tool from NCBI database. Out of which, the best homology (template/reference) protein sequences were selected based on the different criteria such as maximum score (580), query coverage (100%), identity (99%) and e-value (0.0), and selected protein sequences werre used for the further analyses. The computation of amino acid composition of query protein sequence using ExPASY's ProtParam tool detected very high percentages of isoleucine (7.5%) and leucine (11.4%) as compared to other amino acids (Table 1) of this protein. The high percentage of those amino acids influences the regulation of signaling pathway and protein synthesis independently [24]. Glycine content shown low percentage (4.3%) and it indicates the less stability in the triple helical structure. The proline residues are equally essential like glycine residues for the helix stabilization of secondary structure in a protein. In this study, the percentage of proline residue was 5.0%, which is less than 10%. That is, it is less efficient for protein stabilization. In the hydrophobic group isoleucine is 16.67% and leucine is 25.40% and with respect to hydrophilic group serine is 20.0% and glutamine is 17.89% plays a key role for protein threedimensional structure. The ExPASy's ProtParam online tools shown in Table 2 were used to compute the physiochemical properties of the query protein. The pI value was 6.15, which is less than 7, so the query protein is acidic and insoluble in dilute mineral acids [25]. The acidic protein has the functional role for the gene expression in the cellular processes [26]. The instability index was greater than 40, so it might be stable in the wet lab experiment. The aliphatic index was 96.48 that are very high. So, the query protein is regarded as the thermostable protein. It means that the query protein C15orf41 is a resistant protein due to the irretrievable changes of physical and chemical structural decay in the high-temperature. The GRAVY value was -0.232, which lies between -2 to +2. It indicates that the query protein is positively rated and more hydrophobic [27]. So, it reduces the contact region between water and non-polar molecules and exploits the hydrogen bonding of water molecules in the cellular process. The domain structure of the query protein shown that its unknown function of TPD (Treponema Pallidum) domain [28] is denoted from 135 to 265 residue positions (Figure 2A) with the evalue= 3.05e-81. It is a family of eukaryotic proteins of unknown function. A few members of TPD domain were associated with zinc-finger domain and it carries an exceedingly conserved TPD sequence-motif. The PTM sites of the query protein were predicted using the online SMART bioinformatics tool. The functional formation of three PTM sites are (i) Acetylation is an important PTM site responsible for the several cellular and biological processes of diseases [29], (ii) Phosphorylation plays a vital role for metabolism, division, organelle trafficking, membrane transport, immunity in the cellular process [30] and (iii) Ubiquitination involved in the 3-steps metabolism process such as ubiquitin-activating enzyme (E1), ubiquitin-conjugating enzyme (E2) and ubiquitin-ligase enzyme (E3) with the internal lysine as a substrate molecule through isopeptide bond [30]. Also it should be noted here that the query protein also exists in the mouse body having the same three PTM sites as early mentioned as details described in the Table 3. The bioinformatics tool (ClustalW in MEGA5.0) was used to align the query sequence with the template/reference sequences. This MSA results was shown in Figure 2B. All the similarities and dissimilarities of aligned sequences with query sequence were highlighted using the offline tool GENEDOC. The phylogenetic analysis of the query protein was performed with the reference proteins using offline MEGA5.0 bioinformatics tool. It was used to find the pattern/group of the query sequence using the neighbor-joining (NJ) algorithm. The best functional similar proteins with the query protein were isoform-1 (gi|194018542|) and open reading frame isoform-CRA_c (gi|119612744|) of Homo sapiens located at chromosome 15 (Figure 3A). The secondary structure of the query protein sequence was predicted using the online bioinformatics tool SABLE (Figure 3B). It was shown that Halpha helix of model-1 & model-2 is dominated loop. Then the query protein becomes unstable for a certain biological function in the cellular process. Then its 3D structure (Figure 3C) was predicted using the online bioinformatics tool 'SWISS-MODEL'. It showed 4 α-helix, 4 β-sheets and few random coils with resolution 3.10 of X-RAY crystallography in both N and C terminal. The PPI networks were performed using online bioinformatics tool namely STRING (Figure 3D). Its shown that the interacting proteins with query protein are ATPBD4 (ATP binding domain4), C15orf53 and FAM98B with similarity scores 0.725, 0.624 and 0.623 respectively. The best interacting protein was human ATP (Adenosine Triphosphate) binding domain4 (ATPBD4) having similarity score 0.725. Phylogenetic analysis of query gene sequence was performed with the reference gene sequences using the e!Ensembl online bioinformatics tool. We had shown that the query protein is most similar functional characteristics with the Gorilla (C15orf41) and Chimpanzee (C15H15orf41) (Figure 4). And it was also shown that the protein domain structure is most conserved domain with the best homology protein and the collapsed alignments are gap 0-33% aligns sequence, apple green 33-66% and green 66-100% align sequence.

## Conclusion

In this study, suggests that the physiochemical properties of the query protein seem to be thermostable, unstable and acidic. We found that the query protein has three PTM sites such as K50 (Acetylation), T114 (Phosphorylation) and K176 (Ubiquitination). The functional similarity is the isoform-1 and isoform-CRA_c of Homo sapiens, Gorilla (C15orf41) and Chimpanzee (C15H15orf41) with the query protein based on the phylogenetic analysis. The study protein showed the similar functional behavior compare with the known proteins functionality of Gorilla and Chimpanzee. The human ATPBD4 is the high interacting score protein with the query protein. This computational study would be helpful for the researchers/scientists/biologists to characterize the other uncharacteristic proteins.

## Competing Interest

The authors declare that they have no competing interests.

## Figures and Tables

**Table 1:** Amino acid composition of uncharacterized protein C15orf41

Amino Acid (AA)	AA	No. AA	AA (%)	Hydrophobic Group (%)	Hydrophilic Group (%)
Ala	A	18	6.4	14.29	
Arg	R	13	4.6		
Asn	N	10	3.6		10.53
Asp	D	15	5.3		
Cys	C	9	3.2		9.47
Gln	Q	17	6		17.89
Glu	E	18	6.4		
Gly	G	12	4.3	9.52	
His	H	14	5		14.74
Ile	I	21	7.5	16.67	
Leu	L	32	11.4	25.4	
Lys	K	14	5		
Met	M	3	1.1	2.38	
Phe	F	10	3.6	7.94	
Pro	P	14	5	11.11	
Ser	S	19	6.8		20
Thr	T	9	3.2		9.47
Trp	W	4	1.4		4.21
Tyr	Y	13	4.6		13.68
Val	V	16	5.7	12.7	

**Table 2:** Physiochemical properties of uncharacterized protein C15orf41

No. AA	Molecular weight	pI	#NAME?	#NAME?	Extinction Coefficient	Instability index	Aliphatic index	GRAVY
281	32263.9	6.15	33	27	41870	47.06	96.48	-0.232

**Table 3:** PTM sites of human uncharacterized protein C15orf41

Sequence Position	PTM Sites	Human Sequence Fragment	Mouse Sequence Fragment
K50	Acetylation	IFSQEYQkHIKRTHA	IFSQEYQKHIKRTHA
T114	Phosphorylation	FLQEHEEtPPSKSII	FLQGHEQTPPSKSVI
K176	Ubiquitination	LRDLLLEkNLSFLDE	LRDLLLKKNLSFLDE

## Abbreviations

TPD: Treponema Pallidum
PTM: post translational modification
3D: three-dimensional
MSA: multiple sequence alignment
PDB: Protein Data Bank
pI: isoelectric point
GRAVY: Grand Average of Hydropathicity
NJ: Neighbor Joining
PPI: protein-protein interaction
ATPBD4 - ATP: (Adenosine Triphosphate) binding domain 4
